# Sickness and the Social Brain: Love in the Time of COVID

**DOI:** 10.3389/fpsyt.2021.633664

**Published:** 2021-02-22

**Authors:** Caroline J. Smith, Staci D. Bilbo

**Affiliations:** Department of Psychology and Neuroscience, Duke University, Durham, NC, United States

**Keywords:** social behavior, infection, sex differences, social stress and social support, immune

## Abstract

As a highly social species, inclusion in social networks and the presence of strong social bonds are critical to our health and well-being. Indeed, impaired social functioning is a component of numerous neuropsychiatric disorders including depression, anxiety, and substance use disorder. During the current COVID-19 pandemic, our social networks are at risk of fracture and many are vulnerable to the negative consequences of social isolation. Importantly, infection itself leads to changes in social behavior as a component of “sickness behavior.” Furthermore, as in the case of COVID-19, males and females often differ in their immunological response to infection, and, therefore, in their susceptibility to negative outcomes. In this review, we discuss the many ways in which infection changes social behavior—sometimes to the benefit of the host, and in some instances for the sake of the pathogen—in species ranging from eusocial insects to humans. We also explore the neuroimmune mechanisms by which these changes in social behavior occur. Finally, we touch upon the ways in which the social environment (group living, social isolation, etc.) shapes the immune system and its ability to respond to challenge. Throughout we emphasize how males and females differ in their response to immune activation, both behaviorally and physiologically.

## Introduction

During this historic moment, humanity is faced with a global pandemic of the novel coronavirus SARS-CoV-2 which causes COVID-19. As a result, we must grapple not only with an enormous infectious challenge, but also with social distancing, isolation, and the fragmentation of social networks. This increased social distance is necessary to prevent viral transmission, but long-term social separation is likely to adversely impact mental health outcomes far into the future. As a highly social species, inclusion in social networks and the presence of strong social bonds is critical to our health and well-being (1–3). Early studies suggest that loneliness and psychological distress have increased significantly during the COVID-19 pandemic as compared to before it began (4, 5) and that this loneliness and perceived social isolation are predictive of increased anxiety, depression, and suicidal thoughts (5, 6). Devastatingly, the populations that appear to be most vulnerable to COVID-19 are also those that bear the greatest burden of psychosocial stress. Specifically, COVID-19 infection and COVID-19-related deaths are highest in minority and low socioeconomic status (SES) populations both in the United States and worldwide (7–10). Importantly, social isolation and social stress—at either the level of the individual or the social group—have been shown to negatively impact immune function, while positive social relationships and higher status within social hierarchies enhance many aspects of immune defense and thus may protect against infection, across a wide array of infectious disease (11, 12). Across the evolutionary continuum, as more complex social structures have evolved, so too has the risk of pathogen exposure. Therefore, immune responses to infection and social systems are inextricably linked. In the context of COVID-19, as well as other infectious diseases, it is critical that we understand the complex interplay between the immune system and the social brain.

Numerous studies from countries including China, Italy, and the United States have found that there is a sex difference in COVID-19 disease prognosis and mortality, with men being more vulnerable than women (13–19). Immune responses to infection, including COVID-19, differ between men and women. For instance, men appear to have higher circulating cytokines such as Interleukin (IL)-8 and IL-18 following COVID-19 infection, while women mount a greater T lymphocyte response (20). These findings are in line with a large body of previous literature demonstrating sex differences in the immune response to a variety of infectious agents, with women typically displaying lower susceptibility to infection, but higher rates of autoimmune diseases [for review see (21, 22)]. In addition, men may be more likely than women to be adversely impacted by social isolation and stress as a result of the pandemic. For example, in a study of 4,000 elderly men and women, loneliness was predictive of mortality at a 10-year follow up in men, but not women (23). Similarly, in a meta-analysis of published studies, Roelfs et al. (24) found that mortality risk is higher under conditions of underemployment and this effect is 37% higher in men than in women (24). In non-human animals, sex differences in social behavior abound, and males and females often differ in their response and susceptibility to social stress. These findings highlight the need to better understand the ways in which sex differences in social behavior and susceptibility to social stress may contribute to sex-specific vulnerability and resilience in the face of infectious agents.

**Figure 1:**
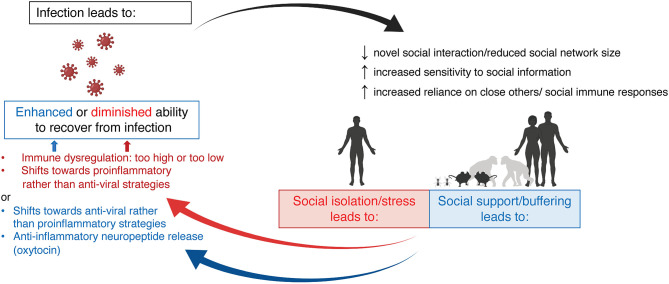
The bi-directional relationship between infection and social behavior. Infection (either viral or bacterial) induces social withdrawal and decreases social network size, across species. Infection also increases sensitivity to social information and increases social interactions with familiar peers and family members. Together, these changes in social behavior aim to reduce disease transmission while promoting individual recovery. Social context can also impact immune function. Social isolation, social stress, or low position within a social hierarchy all lead to immune dysfunction—often shifting immune responses toward pro-inflammatory rather than anti-viral strategies. Conversely, strong social bonds and high social status are associated with a shift in immune responses toward anti-viral strategies and higher oxytocin release, which may act as an anti-inflammatory mediator. Created with BioRender.com.

In this review, we will reflect on the bi-directional relationship between social behavior and the immune system, with an emphasis on how it differs between the sexes. First, we will review the acute effects of infection, either bacterial or viral, on social behavior in both humans and other animal species and how these behavioral effects differ between males and females. We will also discuss the neuroimmune mechanisms that have been posited to underlie these behavioral changes. While many studies demonstrate that maternal infection (or immune activation more broadly) during pregnancy and early life infection can impact social behavior later in life, here we will focus largely on acute adult infection for the sake of scope. Next, we will explore the ways in which the social environment (group living, social isolation, social status, etc.) and perceptions of social connectedness shape the immune system and its ability to respond to challenge in males and females across species. Finally, we will touch on the mechanisms by which this social context is encoded in immune function. Together, we hope to highlight that group living and pathogen defense go hand-in-hand, and that neither can be completely understood without consideration of the other Figure 1.

## How Does Infection Change Social Behavior?

Early observations of the behavior of sick animals and humans noted a constellation of behavioral changes that have been termed “sickness behavior.” These behaviors include lethargy, anorexia, and social withdrawal and are not simply negative effects of the pathogen, but critical adaptive responses on the part of the host aimed at recuperating and reducing the spread of infection to other individuals (25, 26). Sickness behavior comes at a cost; lethargy, anorexia, and social withdrawal weaken the individual, increase risk of predation, and limit social opportunities such as in the context of mate selection and parental care (27, 28). Therefore, the display of sickness behavior represents an inherent trade off. Several theories as to the utility of sickness behavior have been proposed. Lethargy likely conserves energy in order to mount and maintain a fever response [critical to fighting off invading pathogens; (25)], while the entire suite of behaviors serves to protect the individuals' kin by reducing physical contacts, decreasing environmental contamination, and signaling illness to other individuals (27). Indeed, several studies have shown that individuals of many species are capable of recognizing, and avoiding interaction with, sick conspecifics (29–32).

### In Humans

In humans, recent studies have provided a nuanced view of the ways in which infection and inflammation alter social behavior [for comprehensive review see (33)]. Much of this work has been conducted in laboratory settings using an experimental challenge with the bacterial mimetic Lipopolysaccharide (LPS). Participants treated in the lab with LPS report increased feelings of social disconnection, loneliness, and social sensitivity as compared to control-treated participants (34–36). These findings are aligned with the idea that individuals withdraw from social contact when ill. However, in some instances, it might be adaptive to approach others during sickness so that they can provide care and support. Inagaki et al. (37) found that participants reported an increased desire to be with close others (spouses or family members) following LPS administration. Similarly, positive social feedback from an unfamiliar peer appears to be more rewarding following LPS as compared to control treatment (38). Eisenberger et al. (33) posits that heightened sensitivity to social information (either positive or negative) following infection may underlie these findings and be adaptive because it facilitates the rapid identification of, and discrimination between individuals who may or may not provide aid during the recuperative process.

### Sex Differences in Humans

Are there sex differences in social withdrawal in humans? Few studies have directly compared social outcomes in both males and females following immune challenge in humans (39). However, Moieni et al. (35) found that females reported higher social disconnectedness scores than males following an LPS challenge. In contrast to females, however, males reported lower subjective social status following LPS administration than at baseline (40). (32) observed that while both men and women complain about symptoms with similar frequency following LPS challenge, men were more likely to emit vocalizations, such as sighs and deep breaths, that might still signal illness to others, than women (32). These findings provide evidence in humans that males and females may manifest changes in social behavior differently during an acute immune challenge. It is important to note, however, that many factors - including those that are often gendered - can influence the display of sickness behavior in humans. Highlighting this, in a separate study, (41) found that how sick participants anticipated becoming predicted how sick they actually became (41). Similarly, in a retrospective self-reported study of sickness behavior, familism, or the valuation of family above the individual, was associated with stronger sickness behavior in men than in women (42). Thus, sociocultural influences may make it difficult to determine biological sex differences in the sickness response in humans.

### In Animal Models

Social withdrawal following an LPS challenge has also been observed in a wide array of non-human animal species Box 1. Vocalizations are used by many species to communicate across social networks and to find and engage with potential mates. Such social contacts are reduced following LPS administration in passerine bird species (54, 55), vampire bats (56), and field crickets (57), among others. Wild barn mice decrease their social contacts and limit the size of their social network following an LPS challenge (58). Similarly, LPS administration to dominant mice promotes hierarchy destabilization in laboratory settings (59). Several studies have found decreases in direct social interaction between novel conspecifics following LPS administration in adult male rats and mice (60–63). Yet, consistent with studies in humans, many instances have been found in which animals prefer or increase social contact following immune challenge—particularly with familiar peers. In rhesus macaques, LPS administration increases time spent engaging in affiliative behavior in both males and females and this effect persists for 24 h after the stimulation (64). Similarly, LPS administration increases social interaction and huddling in male and female rats (65) and enhances partner preference in female prairie voles (66). In vampire bats, LPS administration decreases social grooming between conspecifics, but this effect is minimal for maternal grooming of infants (56). Together, these findings indicate that changes in social behavior following infection are highly context- and social partner- specific. This specificity is likely related to the inherent trade-offs in sickness behavior discussed above. For instance, individuals may limit social interactions with novel individuals in order to reduce disease spread, but maintain, or even enhance interactions with familiar conspecifics to promote self/kin survival. Aspects of the social context, such as whether or not an individual is currently rearing offspring, may shift the risk vs. benefit of suppressing or engaging in sickness behavior, thereby altering the degree to which changes in social behavior are displayed following infection (67).

Box 1 **The relationship between infection and social communities.**

In addition to changes in social behavior at the level of the individual, infection, or the risk thereof, often changes social behavior at the community level. These “social immune responses” are especially common in animal species that live in large, complex social groups where risk of infection and transmission is high, such as eusocial insect species (43, 44). They consist of behaviors aimed at reducing exposure to pathogens in the environment and limiting the establishment and transmission of pathogens once the community has been infected (45). For example, honeybees respond to invasion of the hive by the heat-sensitive fungus *Ascosphaera Apis* which causes “chalkbrood disease” by generating a behavioral fever (rise in hive temperature) which is protective against infection (46). Several insect species, including honeybees and wood ants, secrete antimicrobial molecules or collect antimicrobial resins from the environment which they incorporate into the building of their nests (47, 48). Furthermore, infected individuals are often either forcibly or voluntarily excluded from the colonies to limit pathogen transmission. Indeed, in the ant species *Temnothorax unifasciatus*, worker ants leave the nest to die in isolation following infection, presumably to limit risk to kin (49). Finally, social grooming is used in insect species including ants, earwigs, and honeybees to transfer antimicrobial or immune mediators between individuals and between parents and offspring (50–52). In a wide-scale study of 11 distinct insect lineages (some eusocial and some non-eusocial), Lopez-Uriba et al. (53) used phylogenetic mixed linear models to test whether colony size predicted cellular immune response. They found that cellular immune responses were lower in larger colonies (53). This finding may suggest that behavioral adaptation, rather than increased cellular immunity, is the most critical defense against the increased risk of infection that comes with community living in such insect species.

Notably, while the effects of the viral mimetic Polyinosinic:polycytidylic acid (Poly I:C) on social behavior have been extensively characterized during the perinatal period (68–70), much less is known as to how it effects social behavior acutely in adulthood. Several studies have shown that Poly I:C increases sickness behavior in adult mice and rats, but none of these studies assessed social interaction (71–73). Further characterizations of the effects of viral challenges on acute sickness responses in adults would add greatly to this body of literature.

### Sex Differences in Animal Models

Despite this abundance of studies, there is a paucity of direct comparisons of social responses to acute immune challenge between adult males and females. One study in adult rats found that LPS administration decreased social interaction but increased huddling with familiar cage mates in both males and females, but with stronger effects observed in females (65). In line with this finding, sexual behavior and sexual receptivity are inhibited following LPS injection in female, but not male rats (74). It has been posited that such sickness-induced decreases in sexual behavior may serve to reduce conception and pregnancy while females are ill (75). In contrast, recent studies suggest that males exhibit more sickness behavior than females following adult administration of LPS, the viral mimetic Poly I:C, or influenza viral infection, but these studies did not include the assessment of social behavior [(76, 77), Sharma et al., 2019]. This, along with sex differences in the neural mechanisms underlying changes in social behavior following infection, remains an important area for future research.

## What Are the Neuroimmune Mechanisms Mediating the Relationship Between Social Behavior and the Immune System?

The process by which changes in social behavior are induced following infection requires highly coordinated and brain-region specific neuroimmune interactions [see review by (39)]. Bacterial and viral infections activate the innate and adaptive immune systems in the periphery. A large body of work shows that toll-like receptor activation in innate immune cells triggers the release of pro-inflammatory cytokines such as IL-1β, IL-6, and tumor necrosis factor (TNF) α which then act in social neural circuits to shift behavior (78–80). Below, we review the brain regions and neuroimmune mechanisms which have been shown to be most critical.

### The Prefrontal Cortex, Amygdala, and Mesolimbic Reward System as Key Neural Substrates for Immune-Driven Changes in Social Behavior

The amygdala, prefrontal cortex (PFC), and mesolimbic reward system stand out as neural structures that have been implicated in both the human and animal literature as important neural mediators of infection-induced changes in social behavior. All are core nodes of the “social decision-making network” which regulates social behavior across vertebrate animals (81). Each likely plays a unique role within these networks. As a “salience detector,” the amygdala is critical to the identification and decoding of social stimuli (82). This information is relayed to the PFC, which is critical to social decision-making (83–85), as well as (both directly and indirectly) to the mesolimbic system [consisting largely of projections from the ventral tegmental area (VTA) to the nucleus accumbens (NAc)] to drive behavior—either approach or avoidance (86–88).

Findings in humans suggest that changes in amygdala activity may serve to increase sensitivity to potential social threats when sick. In a randomized controlled trial, LPS administration increased amygdala activation in response to threatening faces as compared to placebo control and this increase was associated with increased feelings of social disconnection (89). Multiple studies have found increased activity of the subgenual anterior cingulate cortex (sACC; a sub-region of the PFC) following immune activation. Specifically, administration of the typhoid vaccine increased activity of the sACC during an emotional face processing task in male participants and increased circulating levels of pro-inflammatory cytokines such as IL-6 (78). Furthermore, the functional connectivity of the sACC with the amygdala, nucleus accumbens, and other regions of the PFC was reduced by this exposure and this effect was mediated by peripheral IL-6 levels (78). Finally, ventral striatum activity is increased in response to images of support figures, but not strangers, following LPS administration in human study participants and this increase is correlated with circulating levels of IL-6 in circulation (37). Together, these studies provide evidence from human studies that the amygdala, PFC, and NAc may be key brain regions in which cytokines act to alter social behavior following infection.

Findings in the animal literature also support the importance of these brain regions as sites of neuroimmune mediation of social behavior. In adult male mice and rats, LPS administration increases the expression of c-Fos (a marker of neural activity) in the amygdala, as well as IL-1β and IL-6 mRNA in the amygdala (90–92). Interestingly, healthy male rats avoid social interaction with conspecifics that have received an LPS challenge, and this effect is dependent on vasopressin signaling in the medial amygdala (93), lending further support for the role of the amygdala in social information processing following infection. LPS administration also increases mRNA for IL-1β, TNFα, and IL-6 in the PFC of adult male rodents (94). As will be discussed in more detail below, microglia, the resident immune cells of the brain are key responders to peripheral cytokines following infection (95). Peripheral LPS administration alters microglial morphology in the PFC (often used as an indicator of function), increasing microglial soma size and decreased process length (96, 97).

Several studies suggest that dopamine signaling in the mesolimbic system may be particularly important for linking infection and social behavior [for complete review see (87)]. Immune challenge has been shown to impact dopaminergic signaling in the context of reward and motivation, albeit not in the social domain (98–100). Activation of VTA projections to the NAc facilitates social interaction (101) and activation of dopamine D1 receptors in the NAc increases social play behavior (102). Work from our laboratory recently showed that in healthy male (but not female) rats, microglial, complement-dependent phagocytosis of D1 receptors in the NAc is required for the normal developmental decline of social play behavior between adolescence and adulthood (103). Intriguingly, Ben-Shaanan et al. (104) found that chemogenetic activation of dopaminergic projections from the VTA to the NAc increased social interactions with familiar cage mates and improved the innate immune response to E. coli infection—decreasing bacterial load (104). This finding is in line with those from human studies suggesting that activation of the ventral striatum (of which the NAc is a component) may increase social interactions with familiar peers, and thus, buffer against infection. Of note, however, some findings are counter to this hypothesis, suggesting that dopamine may increase neurotoxicity following infection (87, 105).

### Immune Mediators of Social Behavior

As evidenced by the studies discussed above, increases in the pro-inflammatory cytokines IL-1β, TNFα, and IL-6 are often associated with changes in social behavior following immune activation and/or with activity in social circuits in the brain (37, 78, 90–92, 94, 106). Several studies provide a causal link between these cytokines and social behavior. For example, in adult male rats and mice, peripheral or central administration of an IL-1 receptor antagonist attenuates the suppressive effects of LPS or IL-1β on social behavior (61, 107, 108). Interestingly, IL-1 receptor blockade also reduces the effects of TNFα on social behavior (79, 80), suggesting synergism between these cytokines. Finally, central administration of either LPS or IL-1β fails to induce social withdrawal in IL-6 KO mice (63). Cumulatively, these studies provide compelling evidence for a causal role of these cytokines in the induction of social withdrawal following infection.

Microglia, the resident immune cells of the brain, are also important mediators of the relationship between infection and social behavior. Peripheral LPS injection increases IL-1β and TNFα mRNA in microglia (109–111). Since LPS does not cross the blood brain barrier, it is likely that local cytokine release by microglia at least partially mediates the impact of immune activation on social behavior. In support, aberrations in microglial function, including chemogenetic manipulation and elevated protein synthesis, disrupt social behavior, and prevent immune activation-induced changes in social behavior (112–114).

Finally, lymphocytes have also been implicated in the neural control of social behavior. SCID mice, which lack mature B and T lymphocytes and are thus deficient in adaptive immunity, have deficits in social behavior that can be restored by lymphocyte repopulation (115). Moreover, mice deficient in interferon-γ (produced by T cells) display social deficits (115). Interestingly, both SCID mice and IFN-γ knock-out (KO) mice also display hyperactivity of the PFC in response to social stimuli. This finding is well-aligned with the human studies demonstrating increased activity of the PFC in response to social information following infection.

### Sex Differences in the Neuroimmune Mechanisms Mediating Social Behavior Following Infection

The vast majority of the work detailed above was conducted only in male animals. However, the studies that have been performed in both sexes indicate potential avenues for further investigation. In humans, in a double-blind, placebo controlled clinical trial on the relationship between cytokines and social behavior, LPS administration increased both circulating IL-6 and TNFα, as well as depressed mood and feelings of social disconnection in male and female participants (35). Interestingly, cytokine increases were greater in females than in males and behavior correlated with cytokine measures in females only (35). In female, but not male rats, IL-1β inhibits sexual behavior (116). In mice, Sharma et al. (73) found sex differences in cytokine mRNA expression in the brain following LPS challenge in adulthood, with males having more IL-1β mRNA expression and females having higher TNFα mRNA expression; however social behavior was not assessed.

In many of the studies discussed earlier, females exhibited greater social behavior changes following infection than males (40, 65, 74). One possibility is that this might reflect better behavioral adaptation in females. In general, males tend to fair worse in the face of infection [for review see (21, 22)], while females tend to mount greater adaptive immune responses to viral infection—as is the case in COVID-19 (19, 20). One obvious mechanism by which sex-specific susceptibility/responsivity might be generated is via sex differences in steroid hormone exposures. In line with this idea, both androgens and estrogens have been shown to influence immune function (117), with broad theories suggesting that testosterone is immunosuppressive while estrogen enhances immune function (118, 119). It has also been proposed that exposure to androgens, in either males or females, may increase susceptibility and variability in responses to challenges (120), lending itself to greater male vulnerability. Less is known regarding the specific role of steroid hormones in *social behavior* changes following infection. However, evidence supports their possible involvement. For example, IL-1β administration increases anxiety-like behavior in female rats in estrus, but not those in non-estrus (121). Moreover, IL-1β administration also increases anxiety-like behavior in ovariectomized females treated with progesterone (121). During healthy brain development, VanRyzin et al. (122) recently showed that testosterone drives increased microglial phagocytosis of newborn neurons in the male brain, contributing to the masculinization of social play behavior (122). Further research is needed to determine the contribution of steroid hormones to changes in social behavior following an acute infection in adulthood.

Microglia represent another particularly attractive candidate to mediate sex differences in the impact of infection on social behavior. Indeed, microglial biology is replete with sex differences during both homeostatic and disease conditions (22, 103, 122–124). For instance, recent work from our lab using RNA sequencing demonstrated that microglial gene expression and morphology differs between males and females at baseline (125). Furthermore, based on a novel “microglial developmental index” based on gene transcription, adult female microglia appear to be more mature than male microglia and acute LPS challenge accelerates microglial development in males only (Hanamsagar et al., 2018). Intriguingly, we have also recently found that microglial pruning of dopamine D1R receptors in the NAc is critical to the normal development of social play behavior in males, but not females (103). It is therefore possible that sex differences in the impact of immune stimulation on microglial function is a route by which sickness leads to sex-specific behavioral responses.

## How Does Social Context Shape Immune Function?

Group living inherently increases individual exposure to pathogens and parasitism, but also provides opportunities for the evolution of collective social responses to protect against infection/parasitism. A large body of literature across species suggests that social isolation and social stress may impair immune function, while strong social bonds may buffer against infection.

### Social Isolation and Immune Function

Early research into the effects of social isolation on immune function revealed striking decreases in pathogen resistance in isolated animals. For example, mice that were housed in individual cages reached 85% mortality following West Nile Virus infection, as compared to only 50% in socially housed mice (126). This was driven by enhanced viral proliferation and mass loss of the spleen and thymus in isolated mice. IL-6 expression in response to either the viral mimetic Poly I:C or LPS is increased in isolated mice as compared to those that were socially housed (71, 127). Social isolation also increases the blood trafficking of leukocytes and monocytes (128) and decreases anti-inflammatory IL-10 mRNA and protein in the blood and brain (129). Wound healing is impaired to a similar extent in male and female mice following social isolation (130, 131), indicating some similarity between the sexes in this outcome.

Findings in humans echo this animal work. In an extreme example, social isolation during space flight or terrestrial preparation for space flight, led to damped immune responses to viral infections and reactivation of latent viral infections such as herpesviruses (132). More frequently, isolation is assessed based on loneliness or “perceived social isolation” in humans. In psychiatric inpatients, reported loneliness was found to be associated with lower immunocompetence (133). Healthy participants (male and female) who reported greater loneliness mounted a greater TNFα, and IL-6 response to an acute LPS challenge than those that did not (134). Similarly, trait sensitivity to social disconnection is associated with a greater inflammatory response (as evidenced by TNFα and IL-6) to LPS challenge, (35). On the other hand, in a recent study of almost 9,000 adults over the age of 50, social engagement and cohabitation were associated with lower levels of pro-inflammatory factors including fibrinogen, C-reactive protein, and white blood cell count, irrespective of sex (135). Thus, whether increases or decreases in inflammatory markers is observed likely depends very much on the specific context and endpoint in question. Furthermore, too little or to great an immune response can have detrimental consequences for the ability to overcome infection. For instance, excessive cytokine release can increase mortality following infection by damaging host tissues, as is the case of the “cytokine storm” observed in some patients with COVID-19 (136).

Many of the same neural structures that mediate the effects of infection on social behavior, also mediate the effects of social context on immune function. PFC gene expression analyzed postmortem indicated that loneliness in the 5 years anti-mortem was associated with an enrichment for immune related genes (137). Greater feelings of loneliness are associated with greater ventral striatum activity (138) suggesting convergence of inflammation and social isolation effects on the ventral striatum. Blocking opioids with the opioid receptor antagonist naltrexone increases feelings of social disconnection (138), suggesting that opioids may play a role in these effects.

### Social Stress and Immune Function

A wealth of studies has shown that social stress, in the form of social defeat stress in rodents, low social rank within a hierarchy in non-human primates, and low socioeconomic status in humans, has severe negative consequences for immune function [for excellent reviews see: (139–141)].

In brief, in male rodents, social defeat increases pro-inflammatory cytokines including IL-6, TNFα, and IL-1β, but decreases the anti-inflammatory cytokine IL-10 in the brain (94, 142–144). Social defeat also exaggerates the impact of an immune challenge with LPS on cytokines production, microglial activation, and monocyte infiltration to the brain (145). Of particular relevance to the current COVID-19 pandemic, lung inflammation is also increased in mice exposed to social stress (146). Until recently, social defeat paradigms were used almost exclusively in male animals, for the simple reason that it was harder to elicit aggression in females. However, recent work has overcome this hurdle by using DREADD technologies to induce aggressive behavior toward females in male rodents (147). In this study, the authors found that social defeat induced similar increases in pro-inflammatory cytokines and monocyte infiltration into the brain (147), albeit without direct comparison between the sexes. Still, it represents an important step toward the inclusion of females in studies of adult susceptibility to social stress.

In primates that live in hierarchically organized social groups, several studies suggest that social status shifts immune function. In female rhesus macaques, the effects of LPS administration on pro-inflammatory gene expression are higher overall in low-ranking vs high-ranking individuals. Furthermore, gene expression patterns are shifted such that low-ranking females up-regulated genes related to bacterial defense, while high-ranking females upregulated more genes related to viral defense (148, 149). The same group has also shown that in wild male and female baboons, males up-regulate many more genes than females in response to LPS and that some of these genes include those that were up-regulated in low-ranking females (150). It is important to note however, that the authors determined that in males, immune gene transcription was a precursor to social status, suggesting that immune function may contribute to social rank (150). This is in line with the idea of a “conserved transcriptional response to adversity” (CTRA) in which adversity biases gene expression toward pro-inflammatory gene expression and away from anti-viral and antibody production genes (151, 152). Similar CTRA gene expression shifts have also been demonstrated in leukocytes from humans exposed to social stress, i.e., low socioeconomic status (153, 154). In a study of peripheral cytokine expression and cognitive function following a flu vaccine in human (largely female) volunteers, participants who had experienced early life social stress displayed more strongly associated changes in IL-6 and depressed mood (155), providing evidence in humans for long lasting effects of social stress on immune responses.

Neuroimaging studies suggest that the PFC and amygdala may be critical to the effects of social stress on immune function. Lower perceived social status is associated with an increased pro-inflammatory response to a social evaluation stress test and greater dmPFC activity during negative social feedback (38). In a study of young women, this social stress task increased serum cytokine levels, amygdala activity, and functional connectivity between the amygdala and the dorsolateral PFC (156). Moreover, this increased connectivity was associated with increased feelings of social rejection (156).

### Social Buffering and Immune Function

While social isolation and social stress potentiate inflammatory responses, social bonds and supportive social networks can also have powerful stress buffering and anti-inflammatory functions (157, 158). In social species, strong social bonds decrease stress and enhance immune function (159, 160). For example, socially monogamous prairie voles exhibit stronger immune responses than socially promiscuous meadow voles (161). In infant Bonnet macaque monkeys exposed to maternal separation, the presence of juvenile conspecific (friend) prevented mitogen-induced increases in leukocyte activation (162). Furthermore, the frequency of affiliative interactions with this companion were positively associated with natural cytotoxicity (162). Similarly, in a study of adults who experienced low socioeconomic status (SES) as children, those who reported high levels of maternal warmth exhibited lower IL-6 responses following stimulation of peripheral blood mononuclear cells [PBMCs; (163)]. Finally, in a study of adult men, perceived social support at home was associated with higher levels of natural killer (NK) cells and a higher INFγ/IL-4 cytokine ratio (164).

As discussed earlier, activation of the mesolimbic reward system, and dopamine signaling in particular, may represent a potential mechanism by which positive social interactions might boost immunocompetence (87, 104). Another potential candidate is the oxytocin (OT) system. Oxytocin is a highly evolutionarily-conserved neuropeptide that mediates social behavior and is released during a variety of social encounters (165). Of particular interest here, OT appears to have anti-inflammatory capacities as well (166, 167). In a randomized controlled trial in adult men, intravenous oxytocin administration blunted LPS-induced increases in a number of immune molecules, including TNFα (168). In singly housed female Siberian hamsters, stress impairs wound healing, but this effect is absent in socially housed hamsters or singly housed hamsters treated with OT (169). Furthermore, OT receptor antagonism delayed wound healing in socially housed hamsters (169). In male mice, *in vivo* systemic treatment with LPS increased TNFα and IL-1β in the PFC 24 h later, but this increase was attenuated by intranasal OT administration (Yuan et al., 2016). *In vitro*, OT also dampened the response of both primary microglia and BV-2 cells to LPS treatment (Yuan et al., 2016). Together, these studies provide evidence in both male and female animals that OT is anti-inflammatory and, thus, of great interest to understanding how social support systems may buffer against immune challenge (158). Importantly, however, direct comparisons between the sexes were not made in these studies. Many studies have shown that OT is an important regulator of social behavior in both males and females (170, 171). Yet, the OT system is also replete with sex differences (172, 173). OT and OTR expression are regulated by steroid hormones (174–176). Therefore, it is highly likely that sex differences in the OT system might contribute to sex differences in the degree to which social interactions can provide buffering in the face of immune challenge.

## Critical Periods and Chronic Illness: the Breakdown of Adaptive Responses

Shifts in social behavior are a critical part of the adaptive host response to infection. However, when immune challenges occur during developmental critical periods or lead to chronic inflammation, they can have long-lasting and maladaptive consequences for social dysfunction—even after the acute illness has passed (95, 177). Immune challenges during the perinatal period disrupt adult social functioning in both males and females, but these effects appear to depend on a variety of factors including developmental timing of the challenge, drug dose, and the nature of the challenge itself (30, 68, 69, 111, 178, 179). For instance, maternal immune activation with influenza virus (70) or Poly I:C during pregnancy leads to social deficits in adult offspring in both rodents and primates (68, 70, 180–182). Several studies have investigated the effects of Poly I:C administration during early to mid-gestation on social behavior in both male and female offspring and observed social deficits only in males (180, 183). Interestingly, late gestational Poly I:C (on the last day of pregnancy) induces social deficits in both male and female offspring (184), which may suggest that sex-specific vulnerability is sensitive to gestational age. Similarly, in rodents, administration of a low dose of LPS between postnatal days (PND) 3–5 has been shown to decrease social behavior in both males and females in adolescence (30, 178) and only in females in adulthood (111), while a high dose of LPS administered at PND 9 only decreases social behavior in adulthood in males (69). Thus, either a viral or bacterial infection may alter social behavior in males and females, but the magnitude of these effects differs between the sexes and changes along developmental trajectories.

Adolescence is a developmental phase during which social motivation is heightened and social interactions are particularly important for the development of appropriate adult social behaviors. It is also a period of sensitivity to immune challenges (185, 186). Several recent studies have taken great strides toward characterizing sex differences in neuroimmune interactions during adolescence as well as their implications for social behavior (73, 76, 103, 187, 188). It appears that while males and females respond differently to immune challenges during adolescence, the pattern of sex differences is often specific to a given neuroimmune endpoint. In line with this idea, Cai et al. (76) found that females mounted greater IL-1β and IFN-γ responses to an LPS challenge than males during adolescence but this was the opposite during adulthood (76). In contrast, IL-6, IL-12, and TNFα responses are similar between males and females during both adolescence and adulthood (76). Finally, male microglia appear to be more amoeboid and less ramified than female microglia in the PFC during adolescence (187). This “activated” morphology is often used as a proxy (albeit a limited one) for microglial functional state as it is observed following immune challenge and correlates with pro-inflammatory cytokine expression (125, 189, 190). Similarly, in the NAc, microglia play a critical role in sculpting social circuits in males but not females (103).

Sex differences in susceptibility to social isolation and social stress are also observed during the adolescent period. In humans, adolescent girls are more likely than adolescent boys to be sensitive to social stress, become depressed, or to engage in self-injurious behavior following stress in peer and/or family relationships (191, 192). In adolescent male mice, social isolation during adolescence leads to impaired social recognition memory in adulthood (193). Furthermore, chronic social stress during adolescence leads to social deficits that are transmitted to the next generation as well (194). In Syrian hamsters, deprivation from social play during adolescence increased social avoidance following social defeat in both males and females but had opposite effects in males and females on aggressive behavior during the social defeat exposure itself (195).

These findings highlight the long-term impact of infection and social stress on social behavior when these challenges occur during developmental critical periods. In these cases, changes in social behavior shift from being acute adaptive responses to an immediate context, but rather a developmental organization of neuroimmune interactions and behavior that can become maladaptive.

## Conclusions: the Importance of Social Context When It Comes to COVID-19

In conclusion, we have highlighted the intimate relationship between immune function and the social landscape. Infection can shift social behavior either for the benefit of the host or for the invading pathogen. Similarly, social context can make individuals either more vulnerable to infection, as in the case of social isolation or psychosocial stress, or it can provide social buffering and promote resilience, as in the presence of strong social bonds. As the scientific community, and the world at large, works to promote resilience in the face of COVID-19, social context must be a major consideration. Furthermore, our synthesis of the literature on infection and social behavior suggests that sex differences in this relationship *in adulthood* remain vastly understudied. Overall, both males and females respond to infection and social isolation, but the few studies that have been conducted suggest nuanced sex differences in the nature of these responses. Given the male bias in susceptibility to COVID-19 and other infectious diseases, it is critical that we understand the sex differences in neuroimmune function that may impart vulnerability and protection to males and females, respectively. Age at infection is also of critical importance. Indeed, it is possible that COVID-19 infections in children that elicit even a mild immune response could have long-lasting impacts on social circuit development. Finally, we must emphasize the ameliorative power of social connection and work to better understand and promote those connections in this era of social distancing.

## Author Contributions

CS and SB conceived of and wrote the manuscript together. All authors contributed to the article and approved the submitted version.

## Conflict of Interest

The authors declare that the research was conducted in the absence of any commercial or financial relationships that could be construed as a potential conflict of interest.
